# Symptoms That May Lead to the Police Court

**Published:** 1914-06-13

**Authors:** 


### THE SCHOOL DOCTOR AND SKIN DISEASE.

### Symptoms that may Lead to the Police Court.

A knowledge of skin diseases is of special value to the school doctor, since there are many conditions in which such knowledge is likely to prove of the greatest help. The ordinary exanthemata are relatively easy to diagnose, and no examiner will find any great difficulty in coming to a conclusion with regard to an anomalous rash, since in such cases the school ought to be given the benefit of whatever doubt exists and the child excluded. Scabies presents some difficulties, especially in a poor-class school and among dirty children. It is not always easy to make an exact diagnosis between this condition and lichen urticatus. The periodicity of the papular rash in the latter may be helpful, but scabies may and does recur periodically. A school doctor has recently, in *School Hygiene*, recorded an epidemic of recurrent scabies in a school under his supervision. Where more than one member of the family is afflicted with the rash, it is exceedingly unlikely that the correct diagnosis is lichen urticatus. At one school the doctor excluded four members of the family, in different departments, who were all suffering from a papular rash on the hands and feet. A provisional diagnosis of scabies was made, and the mother advised as to treatment. She took the eldest child to the out-patient department of a general hospital, where she was informed that the child was suffering from a blood rash and was fit for school. The hospital doctor, however, refused to give a certificate, and consequently the child was again seen by the school doctor, who again diagnosed scabies. On investigation it was found that two adult lodgers in the same house also suffered from the rash, which cleared up on appropriate treatment.

A more interesting condition of the skin, from a school doctor's point of view, is dermographism. While it has, perhaps, no clinical importance, this condition may sometimes give rise to difficulties and may even land a teacher in the police court. Children with this type of skin show any marking they may have through slight trauma to an extent which has to be seen to be believed. It is a condition relatively common in weakly, asthenic children, and occurs in certain definite types. According to R. Müller, who has made a special study of the condition (*Deutsche Zeitschrift f. Nervenheilk.*), four well-marked types are met with: (1) Dermographia alba, in which slight trauma, such as rubbing the skin, or rapidly drawing the finger across the child's arm, leaves a white mark; this depends on nervous excitability of the capillary walls, and is frequently met with. The mark does not persist for more than a few minutes and is succeeded by an equally transient erythema; (2) Dermographia rubra; in this the same slight trauma evokes a brilliant red reflex which lasts a little longer than the white mark and then gradually fades. In neither condition is a definite wheal present, even when the trauma has been comparatively severe; (3) Reflex erythema, which is much rarer in children than in adults and is nearly always a sign of nervous irritability. It is seen especially frequently in children with infantile paralysis and latent Basedow's disease, and any child in whom it is met with should be carefully examined; (4) Dermographia elevata; this is the urticaria factitia of older writers and is by no means uncommon, in its milder forms, in children. In such children a comparatively slight trauma at once raises a heavy red wheal which may persist for hours, sometimes even for days. It is such a case that may land the teacher in difficulties. A child may be corporally punished, by no means severely, but such a child may have a "dermographic skin" (the tautology is inevitable), and the punishment may leave marks quite out of all proportion to its real severity. The parents or friends of the child may notice these marks and reasonably suppose that the punishment has been excessive and brutal. The family practitioner, examining the child and not knowing the skin condition, may agree with this supposition and the case may come up for the decision of the magistrate.

### The School Doctor's Evidence.

This latter arbiter knows nothing of the subtleties of dermographia elevata, but believes the evidence before him and takes it for granted that punishment, to have left such wheals, must have been extraordinary. As a matter of fact it may have been nothing of the kind, for close examination will show that the cuticle is nowhere broken, and that the wheal is in no sense similar to that caused by a ferocious stroke of the cane. It is in these cases that the evidence of the school doctor will be valuable, and it is always worth while to point out the condition, when discovered at the routine examination, to parent and teacher and to touch upon the mistakes that may occur.

Seborrhœic lesions in children are mostly found on the scalp. Sometimes they may be extremely circumscribed and yet severe, so that the scales are heaped up in a small mass. Where an impetiginous condition exists, owing to the child scratching the place, this mass may be still further heaped up and matted with the hair, so that the case resembles one of favus. It is a moot point whether these children should be excluded or not. Since hair-brushes are not used in schools ordinarily, the direct danger of infection seems to be small and can easily be guarded against. But it is always preferable to exclude such children temporarily, firstly, to give them ample time for treatment; and, secondly, to get rid of the psychical impression which these unsightly heads make upon other children in the class. Careful attention to treatment generally soon makes the child presentable once more; a permanent cure is usually more difficult.

